# A Doctor's Narrow Escape

**Published:** 1888-09

**Authors:** 


A DOCTOR'S NARROW ESCAPE.—Dr. William Knight, professor of anatomy at the Ohio Dental College, had his arm nearly eaten off by a black bear at the Cincinnati Zoological Garden recently. He had thrust his hand through the bars of the cage to give the bear some peanuts, when the brute seized his wrist with its teeth and bit the hand almost off.

